# Repeated Psychosocial Screening of High School Students Using YouthCHAT: Cohort Study

**DOI:** 10.2196/20976

**Published:** 2020-10-26

**Authors:** Hiran Thabrew, Harshali Kumar, Mary Goldfinch, Alana Cavadino, Felicity Goodyear-Smith

**Affiliations:** 1 Department of Psychological Medicine The University of Auckland Auckland New Zealand; 2 Tamaki College Auckland New Zealand; 3 Department of Epidemiology and Biostatistics The University of Auckland Auckland New Zealand; 4 Department of General Practice and Primary Healthcare The University of Auckland Auckland New Zealand

**Keywords:** mass screening, mental health, school health services, eHealth

## Abstract

**Background:**

Psychosocial problems are common during adolescence and can have long-lasting effects on health and on academic and social functioning. YouthCHAT, an electronic HEEADSSS (home, education, eating, activities, drugs and alcohol, suicide and depression, sexuality and safety)-aligned instrument, has recently been demonstrated to be an acceptable and effective school-based psychosocial screener for 13-year-old (Year 9) high school students.

**Objective:**

This study aims to compare acceptability and detection rates with repeated YouthCHAT screenings of high school students when they are 13 years old (Year 9) and 14 years old (Year 10).

**Methods:**

We invited all Year-10 students to complete a YouthCHAT screening in 2018. Rates of positively identified issues were compared between the subset of students screened in both 2017 and 2018. Student acceptability toward YouthCHAT was investigated through focus group sessions. Onward clinical referral rates in 2018 were also investigated to explore the potential referral burden following screening. Data analysis for rates of positively identified issues were conducted with the McNemar test. Chi-square, Fisher exact test, and Kruskal-Wallis test were used to analyze the focus group data.

**Results:**

Of 141 eligible Year-10 students, 114 (81%) completed a YouthCHAT screening during 2018, and 97 (85%) of them completed it for a second time. Apart from depression, which increased (*P*=.002), and perceived life stress, which decreased (*P*=.04), rates of identified issues were broadly similar between 13 and 14 years of age. Repeated screenings via YouthCHAT was acceptable to students and time-efficient (mean, 6 minutes and 32 seconds) but did not reduce the overall number of individuals with identified issues. Onward clinical referrals from positive screens were mostly managed by school-based health services without the need for external referrals.

**Conclusions:**

Although further evaluation is needed, our results support the value of YouthCHAT as an acceptable and effective instrument with which to achieve routine identification of psychosocial issues and early intervention within a high school environment.

## Introduction

Adolescence is a period of great change and new challenges, when young people aged 13-18 years may experience psychological challenges that result in either distress or disorder [1]. These challenges can have a short-term impact on their general health, academic functioning, and relationships, and a longer-term impact on their adult functioning [2-4]. Almost half of adult mental health issues originate during adolescence, and early intervention can make a difference to their long-term trajectories [5,6]. Young people often find it hard to seek help, partly due to limited health literacy and the stigma of attending mental health services [7,8]. Many clinicians have also been found to lack the resources to identify psychosocial problems due to a lack of time, a lack of confidence, and concerns about the over-identification of problems for which personal management skills and access to services may be limited [9]. Although routine screening for psychosocial problems has been recommended for many years [10], until recently, this has not been feasible, primarily due to the lack of a suitable instrument that could identify the range of psychosocial issues faced by young people. Instead, opportunistic screening has been undertaken in schools and clinical settings using cumbersome and psychometrically invalidated face-to-face assessments such as the HEEADSSS (home, education, eating, activities, drugs and alcohol, sexuality, suicide and depression, safety) interview [11].

In New Zealand, routine psychosocial screening of high school students is undertaken using the HEEADSSS assessment. However, due to financial constraints, this is restricted to Year-9 students (13 year olds) in low decile schools [12]. In a typical high school, one or two school nurses take 6-12 months to complete these assessments [12]. Although completion rates are reported to the New Zealand Ministry of Health, outcomes of these assessments are neither reported nor published.

Our research group in Auckland, New Zealand, developed an electronic psychosocial screener called YouthCHAT (Youth version, Case-finding and Help Assessment Tool) to be used with young people (aged between 13-25 years), based on the adult screener CHAT [13] and its electronic version, eCHAT [14]. It comprises 13 modules designed to align with the HEEADSSS interview and includes 3 validated screeners, for anxiety [the GAD-7 (7-item instrument for Generalized Anxiety Disorder)] [15]; depression [PHQ-A (Patient Health Questionnaire–Adolescents)] [16]; and substance misuse, such as smoking, drinking, and recreational drug use [SACS (Substances and Choices Scale)] [17]. Other domains covered by YouthCHAT are problematic gambling, general stresses, behavior problems, eating problems, exposure to abuse, sexual health, anger management problems, and physical activity [14]. For each positive screen within a domain, a help question appears, which asks participants if they would like help today or later. The help question provides an opportunity for young people and their health providers to further discuss any issues that young people may want to address, thereby enhancing mutual decision-making. YouthCHAT is designed to be accessed as a website by health providers who have secure access to it. Once a YouthCHAT screen is completed, a summary report is generated for the health provider to review [12].

A counter-balanced randomized trial comparing YouthCHAT and the HEEADSSS assessment was conducted in a low decile Auckland high school with 13-year-old (Year 9) students in 2017. The study was registered with the Australian New Zealand Clinical Trials Registry (ANZCTR) ACTRN12616001243404p and was approved by the New Zealand Northern Region Ethics Committee (16/CEN/137/AM03). The study found that YouthCHAT was twice as fast to complete as the HEEADSSS assessment, as effective at identifying key psychosocial problems, and acceptable to both students and school nurses [12].

Due to the emergence of psychosocial problems across the whole of adolescence, it is likely that single episode screening, particularly when focused at its onset, will fail to identify the majority of problems faced by young people. We have previously proposed routine school-based screening as part of an annual health check to maximize the chance of early identification of psychosocial problems and normalize psychosocial screening within the context of a holistic health assessment. While YouthCHAT has shown promise as a psychosocial screener, its suitability for repeated use within a high school or other settings is currently unclear. Therefore, we decided to use YouthCHAT to rescreen the same cohort of 14-year-old (Year 10) students who were previously screened with YouthCHAT at 13 years of age in 2017, in the same high school [12]. Due to concerns about the potential burden on health services from positively screened individuals, we also planned to explore the nature of onward clinical referrals required by the group in 2018.

This study was designed to (1) provide novel insights into the acceptability of repeated screenings of high school students using an electronic screener such as YouthCHAT; (2) provide information on differing rates of psychosocial problems between 2 consecutive years (to see if rates increase with age, as might be expected during adolescence, and to see if being positively screened or receiving early intervention in 2017 was associated with a reduction in the severity of symptoms in 2018); and (3) provide information about resulting clinical referrals.

Our specific aims were to (1) compare acceptability and detection rates with repeated YouthCHAT screenings of high school students when they were 13 years of age (Year 9) and 14 years of age (Year 10), and (2) examine where onward clinical referrals were provided to those with identified needs.

## Methods

### Participants

All Year-10 (14- to 15-year-old) students at a low decile high school in Auckland, New Zealand, who had previously taken part in a YouthCHAT study in 2017 in Year 9 (at 13 to 14 years of age) [12] were invited to participate in this study, following the provision of paired informed individual assent and parental consent. No students were excluded. A subset of 20 students was invited to participate in a series of focus groups, during which verbal and written feedback regarding their use of YouthCHAT was obtained.

### Procedure and Instrumentation

Written informed consent from students and paired assent from parents were obtained using paper forms. As some students had left and others had arrived between 2017 and 2018, only a subgroup of the total sample consisted of paired participant data from the same students screened in both years. Differences in acceptability of repeated screenings and changes in rates of identified psychosocial problems were analyzed in this subgroup.

YouthCHAT screening was completed on a tablet device with a Wi-Fi connection to the YouthCHAT website in the school health center over an 8-month period, between April and November 2018. A series of 4 focus groups with between 5-10 participants each was conducted on school premises in November 2018 to obtain written and verbal feedback from a subset of students who had been invited to take part by the school nurse, based on their interest and availability.

Encrypted YouthCHAT results were manually extracted and securely stored on a University of Auckland server as per New Zealand Northern Region Ethics Committee (16/CEN/137/AM03) requirements. Student demographic and postscreening referral data were also manually extracted from Medtech (a locally utilized patient management software system) and securely stored on the University server. Written feedback from focus group participants was collected on paper forms and securely stored on University premises. Audiotaped feedback from focus group participants was transcribed by an agent who had signed a confidentiality agreement, and electronic audio-files were securely stored on the University server.

### Outcomes

Psychosocial problems from YouthCHAT were measured by positive responses to any items. Substance misuse was measured by positive responses to A Stop Smoking In Schools Trial (ASSIST) [18] or the Substances and Choices Scale (SACS). Problems with eating were determined by positive responses to any items in the eating module. Mental health and distress were measured by positive screens to the PHQ-A or GAD-7 scale. Life stresses were measured by positive responses to questions regarding different stresses such as the following: issues at home, school, or work; money; or relationships with specific people in one's life. Sexual health was measured by positive responses to concerns regarding sexual orientation, risky sexual behavior, or exposure to unwanted sex. Safety was determined by positive responses to either the anger or abuse modules, and to being bullied or exposed to violence in the stress module. Physical inactivity was measured by negative responses to regular physical activity engagement.

### Data Analysis

Data were analyzed using Microsoft Excel (version 15.0; Microsoft) and Statistical Package for the Social Sciences (SPSS; version 25; IBM Corp). The YouthCHAT screening was considered positive if any items were acknowledged within each module. The depression, anxiety, and substance misuse modules were further examined to ascertain the severity of the results (rated as subthreshold, mild, moderate, or severe, according to the developers of the PHQ-A, GAD-7, and SACS). Analysis of paired data (for those who completed YouthCHAT in both 2017 and 2018) was conducted using the McNemar test for categorical variables. Focus group data from 2017 and 2018 were treated as independent groups. Responses to items regarding YouthCHAT and its use were analyzed using chi-square or Fisher exact tests to compare categorical variables; the Fisher exact test was used where there were any expected cell counts less than 5 [19]. The Kruskal-Wallis test was used for continuous or ordinal variables.

## Results

### Description of Participants

Of 141 eligible students, 114 (80%) were screened in 2018; incomplete data for 1 screening provided a total sample size of 113 for analysis in 2018 and 129 (93%) out of 139 in 2017. None of the invited students declined to take part in the study. A subset of 97 students who completed YouthCHAT screenings in both years were compared. In regard to participant ethnicity, of the paired subset, 60 (62%) students were Pacific Island, 24 (25%) were Maori, 1 (1%) was New Zealand European, 1 (1%) was Middle Eastern/Latin American/African, and 11 (11%) were of other ethnicities. The number of men (50/97, 52%) and women (45/97, 46%) in the paired subset were relatively similar, with 2 students (2%) identifying as gender diverse (which was an additional option in the 2018 YouthCHAT screener). Of the 20 participants invited to take part in focus groups, 16 agreed to do so. The self-reported demographic details of the 16 students who attended the focus groups were as follows: 5 men (31%), 9 women (56%), and 1 gender-diverse individual (6%); 7 (44%) were of Maori descent, 5 (31%) were of Pacific Island descent, and 3 (19%) were of other ethnic descent. Demographic details for 1 participant are unknown as they did not complete the attendance sheet.

### Time Taken to Complete YouthCHAT and Time Period Between Screens in Paired Subset

The average time taken to complete YouthCHAT in 2018 was 6 minutes and 32 seconds (range, 2 minutes and 13 seconds to 16 minutes and 45 seconds). This was similar to the 8 minutes and 57 seconds (range, 1 minute and 45 seconds to 54 minutes and 15 seconds) taken by students in 2017. The Wi-Fi connection was lost for some students in 2017, which may explain the outlier of 54 minutes and 15 seconds. The average time period between the YouthCHAT screens in 2017 and 2018 was 11.4 months (range 6-18 months), with the time periods varying for each student from the paired subset.

### Overall Detection Rates and Comparison of Results in a Paired Subset Between 2017-2018

Overall rates of detection of psychosocial problems and a comparison of results between 2017 and 2018 are presented in Table 1. The table displays the number of students who were positive for psychosocial problems in both years and the number of students who remained positive for the particular psychosocial problem in the repeated screening. It also represents the number of new positive screens for psychosocial problems in the following year of YouthCHAT screening. There was a statistically significant reduction in the rate of perceived life stress (-13%; 95% CI -25.1% to -1.0%; *P*=.04), a statistically nonsignificant increase in the rate of substance misuse, and a statistically significant increase in the rate of depression (18.8%; 95% CI 6.8-30.7; *P*=.002) between the 2 years. According to the 2018 PHQ-A scores, of the 96 respondents, 25 students had subthreshold depression, 3 students had mild depression, 4 had moderate depression, 4 had moderately severe depression, and 1 had severe depression. Otherwise, rates of psychosocial problems between these screening events were broadly similar, with concerns about weight and eating, and behavior and anger, being the most reported issues. Of the 90 students who self-identified with anxiety via the GAD-7 in 2018, 7 had subthreshold anxiety, 11 had mild anxiety, 7 had moderate anxiety, and 1 had severe anxiety. In regard to SACS scores, 8 students had scores greater than 2, indicating they required further assessment; 3 had scores greater than 4, indicating clinically significant problems; and 3 had scores greater than 6, indicating serious problems. Of the 5 students who screened positive for depression in 2017 and 2018, 3 had changed from severe to moderately severe symptoms, 1 remained unchanged with mild symptoms, and 1 had changed from moderate to moderately severe symptoms. Of the 8 students who screened positive for anxiety in both 2017 and 2018, 3 had changed from severe to moderate/mild, and 6 remained unchanged with either mild or subthreshold symptoms.

**Table 1:** Change in detection rates between 2017 and 2018 for students who completed YouthCHAT screenings in both years (n=97).

YouthCHAT domain	Total responses, N (%)	Total positive in 2017^a^, n (%)	Total positive in 2018^a^, n (%)	Remained positive in 2018 (% of those positive in 2017)^b^, n (%)	New positive screens in 2018 (% of those not positive in 2017)^a^, n (%)	Difference in proportions positive in 2017 & 2018 (95% confidence interval)	*P* value^c^
Smoking	94 (97)	9 (10)	13 (14)	6 (67)	7 (7)	4.3 (-3.4 to 11.9)	.34
Drinking	92 (95)	5 (5)	10 (11)	3 (60)	7 (8)	5.4 (-2.0 to 12.8)	.18
Drugs	92 (95)	6 (7)	12 (13)	5 (83)	7 (8)	6.5 (-0.4 to 13.5)	.07
Eating disorder	95 (98)	93 (98)	94 (99)	92 (99)	2 (2)	1.1 (-3.6 to 5.7)	>.99
Depression	96 (99)	12 (13)	30 (31)	5 (42)	25 (26)	18.8 (6.8 to 30.7)	.002
Self-harm	93 (96)	6 (6)	5 (5)	2 (33)	3 (3)	-1.1 (-7.7 to 5.6)	>.99
Anxiety	90 (93)	20 (22)	19 (21)	8 (40)	11 (12)	-1.1 (-12.7 to 10.4)	>.99
Stress	92 (95)	29 (32)	17 (19)	9 (31)	8 (9)	-13.0 (-25.1 to -1.0)	.04
Behavior	92 (95)	53 (58)	48 (52)	36 (68)	12 (13)	-5.4 (-17.9 to 7.1)	.46
Sexual health	92 (95)	21 (23)	16 (17)	9 (43)	7 (8)	-5.4 (-15.7 to 4.9)	.36
Abuse	85 (88)	19 (22)	12 (14)	5 (26)	7 (8)	-8.2 (-19.8 to 3.4)	.19
Anger	85 (88)	33 (39)	32 (38)	19 (58)	13 (15)	-1.2 (-14.3 to 12.0)	>.99
Physical inactivity	88 (91)	37 (42)	26 (30)	17 (46)	9 (10)	-12.5 (-25.3 to 0.34)	.06

^a^The denominators (N) are represented in the “Total responses” column.

^b^The denominators for this column vary, as they are the total individuals screened positive to the YouthCHAT domains in 2017.

^c^*P* value from the McNemar test.

### Acceptability of Repeated Screening Using YouthCHAT

In 2018, the 16 students who took part in the focus group gave YouthCHAT a rating of 7.8 (range 5-10) on a point scale from “lame” to “awesome;” they also said it had been helpful for discussing psychosocial issues with their school nurse or doctor at a rating of 7.8 (range 5-10) on a 10-point scale from “not helpful at all” to “helpful.” Although many (10/16, 63%) denied disliking any YouthCHAT questions, 6 of the 16 students (38%) said they did not like answering questions related to sexual health, substance misuse, exercise, anger, gambling, and depression. The results of student rating scores from both 2017 (n=10) and 2018 (n=16) are presented in Tables 2 and 3; they were not paired, as different groups of students attended the focus groups in each year. Overall, there were no major differences in the acceptability of YouthCHAT from one year to the next. Although fewer students said they had time to think about their responses in 2018, there was a notable reduction in feelings of embarrassment about discussing results with the school nurse, and a similar number of students reported comfort with disclosing things they would otherwise have not mentioned in both years.

**Table 2:** Comparison of student acceptability of YouthCHAT in 2017 (n=21) and 2018 (n=16).

Rated item		YouthCHAT in 2017 (n=21), n (%)	YouthCHAT in 2018 (n=16), n (%)	Change 2017-2018, *P* value
Works for people my age		18 (86)	10 (63)	.14^a^
I have time to think about my responses		16 (76)	7 (44)	.04
I felt safe answering the questions		14 (67)	9 (56)	.52
I talked about the things I wouldn't have mentioned		11 (52)	2 (13)	.01
It's easier to open up about my unhealthy behaviors and feelings		13 (62)	12 (75)	.40
It helped me identify the unhealthy behaviors and feelings I need help with		14 (67)	7 (44)	.16
Allowed my nurse to know about the unhealthy behaviors and feelings		13 (62)	8 (50)	.47
Has too many questions		6 (29)	3 (19)	.70^a^
Questions are too personal		5 (24)	1 (6)	.21^a^
I worried about the privacy of my information		9 (43)	3 (19)	.12
Takes too long		4 (19)	4 (25)	.71^a^
Questions were difficult to understand		2 (10)	2 (13)	.37
Questions did not relate to me		1 (5)	0 (0)	>.99^a^
Is boring		2 (10)	3 (19)	.63^a^
I felt embarrassed to talk to my nurse about my answers		6 (29)	0 (0)	.03^a^
My nurse was judgemental about things I opened up about		1 (5)	0 (0)	>.99^a^
**Objected to specific questions**				
	Substance misuse	9 (43)	7 (44)	.96
	Sexual health	8 (38)	6 (38)	.97
	Safety	6 (29)	1 (6)	.11^a^
	Physical inactivity	2 (10)	1 (6)	>.99^a^
	Gambling	0 (0)	1 (6)	.43^a^
	Depressed or low	0 (0)	2 (13)	.57^a^
	Anger control	0 (0)	1 (6)	.43^a^

^a^*P* value for YouthCHAT items from Fisher exact test; the remaining *P* values are from the Pearson chi-square test.

**Table 3:** Focus group ratings of YouthCHAT in 2017 (n=10) and 2018 (n=16).

Rated item on a 10-point scale	YouthCHAT in 2017 (n=10), mean (range)	YouthCHAT in 2018 (n=16), mean (range)	Change 2017-2018, *P* value^a^
YouthCHAT helpfulness: “not helpful at all” to “helpful”	8.2 (5-10)	7.3 (3-10)	.57
YouthCHAT appraisal: “lame” to “awesome”	9.0 (8-10)	7.8 (5-10)	.33

^a^*P* value for YouthCHAT rating from Kruskal-Wallis H test.

### Onward Clinical Referrals From YouthCHAT Screening

Table 4 presents the number of students who were referred to internal school health staff, health services, and programs following the YouthCHAT screening in 2018. Onward clinical referrals were made by the school nurse based on the severity of the psychosocial issue (eg, a concerning score from the PHQ-A questionnaire) and whether school health staff were already seeing the student who screened positive for any psychosocial problems prior to the YouthCHAT screening. As evident from Table 4, most students with positive YouthCHAT screens who received onward clinical referrals following school nurse review of their results were seen by school-based practitioners, particularly nurses and counselors. Only a few required assistance from external community-based or specialist health services. All students referred for onward clinical referrals had positive YouthCHAT screens, and most of those with depression (12/113, 11%), anxiety (19/113, 17%), and substance misuse (11/113, 10%) had clinically significant levels of symptoms.

**Table 4:** Referrals to school staff or youth development services for 113 students screened with YouthCHAT in 2018. (Note: some onward clinical referrals to school health staff or external services overlap due to multiple referrals for some students.)

Psychosocial issue	No onward clinical referral required, n (%)	Referrals to internal school staff			Referrals to external agencies and programs	
		Nurse, n (%)	Counselor, n (%)	Social worker, n (%)	n (%)	Type of referral
Depression	100 (88)	1(1)	13 (12)	N/A^a^	2 (2)	Specialist mental health service
Self-harm	104 (92)	1 (1)	9 (8)	N/A	N/A	N/A
Anxiety	97 (86)		16 (14)	N/A	N/A	N/A
Substance misuse	96 (85)	1 (1)	8 (7)	N/A	11 (10)	Addiction program
Sexual health	89 (79)	11 (10)	17 (15)	N/A	N/A	N/A
Eating/ exercise	95 (84)	1 (1)	3 (2.6)	N/A	15 (13)	Fitness program
Behavior concerns	95 (84)		17 (15)	N/A	1 (1)	Behavior support service
Safety	77 (68)	1 (1)	34 (30)	N/A	2 (2)	Anger management counseling agency
Stress	85 (75)		21 (19)	11 (10)	N/A	N/A

^a^N/A: Not applicable.

## Discussion

### Principal Findings

Our results demonstrate that YouthCHAT remains an acceptable, efficient, and effective HEEADSSS-aligned instrument for undertaking repeated psychosocial screenings with high school students. They also suggest that most identified problems can be managed within the school environment. Overall rates of identified issues at 14 years of age were not that different from those identified at 13 years of age, which may be unsurprising given the fact that most significant mental health issues emerge between midadolescence and early adulthood [20]. However, the relatively high rates of anxiety (21%, compared to 13% in a 15-year-old youths sample from a previous New Zealand study) and depression (31%, compared to 3% in 2017 and 6% from the same previous New Zealand study of 15-year-old adolescents [21]) in this study are interesting to note. These may partly be explained by the predominance of students of Pacific Island ethnicity and their known propensity to experience twice the rates of these issues as other New Zealand youth [22]. Reasons for higher rates of these conditions in this group are not fully understood but may bear some relationship to culturally mediated values [23], status incongruity [24], and nonculturally related sociodemographic factors (such as social deprivation) [25].

Many of those identified with depression (42%), anxiety (40%), substance misuse (60-83%), and eating concerns (99%) at 13 years of age also screened positive at 14 years of age. Although it might seem like screening did not make a difference to later rates of these problems, the truth is probably more complex. This was a relatively small sample, and most students who screened positive for depression and anxiety experienced a maintenance or improvement in symptoms, which, in the context of naturally increasing rates and severity of these conditions, may indicate that earlier intervention had some clinical effect. Certainly, larger, more detailed, and longitudinal studies are needed to more accurately evaluate the value of routine screening in reducing the long-term prevalence of psychosocial problems and their associated disability. The fact that most onward clinical referrals were undertaken by school nurses and counselors underscores the role of YouthCHAT in supporting early intervention within students' natural environments. It also assuages the concern that routine screening increases the risk of further burdening stretched specialist mental health services [12].

### Limitations

This study was conducted at the same high school as a previous study so that results between both years could be compared. However, this remained a convenience sample, and the generalizability of study results to other high schools remains to be proved. The inclusion of primarily Pacific Island and Maori students, who comprised the bulk of students in the examined class, is both a strength and a weakness. Although they comprise 11% and 20% of the New Zealand population, respectively, Pacific Island and Maori youth have higher rates of psychological issues, including depression and suicide [22]. They are also usually harder to reach, accessing specialist services at lower rates than other ethnicities [26,27]. Due to funding limitations, feedback was not collected from school nurses or counselors regarding their satisfaction with receiving referrals following repeated YouthCHAT screenings.

### Conclusions

This study demonstrates the acceptability and effectiveness of the repeated use of YouthCHAT for students. The fact that the majority of positive screens to YouthCHAT were manageable within the school health setting supports the feasibility of routine psychosocial screening in the school environment and the likelihood of earlier intervention. YouthCHAT is available for use by schools and can be easily implemented by school nurses and counselors for opportunistic and routine psychosocial screening. Routine screening via YouthCHAT may lead to reduced costs compared with lengthy face-to-face clinical assessments and more timely interventions for students within the school environment.

Recommendations for immediate research include trialing YouthCHAT with older (15- to 18-year-old) students within the same high school to examine age-related trends, and evaluating differing acceptability and rates of psychosocial problems between low and high decile, urban and rural, and English and Maori immersion schools. International studies, with or without culturally relevant adaptation, and longitudinal studies to gauge the impact of earlier intervention following routine screening, would also be worthwhile. For the moment, YouthCHAT remains the only electronic psychosocial screener with evidence of its acceptability and feasibility for repeated use in any type of setting.

## Abbreviations

GAD-7: 7-item instrument for Generalized Anxiety Disorder
HEEADSSS: home, education, eating, activities, drugs and alcohol, sexuality, suicide and depression, safety
PHQ-A: Patient Health Questionnaire–Adolescents
SACS: Substances and Choices Scale
YouthCHAT: Youth version, Case-finding and Help Assessment Tool
